# H&E-Referenced Multiplex Immunofluorescence Interpretation in TMA Cores: Spatial Co-localization, Cell Feature Validation, and Virtual H&E Generation

**DOI:** 10.21203/rs.3.rs-9802601/v1

**Published:** 2026-07-07

**Authors:** Jun Jiang, Raymond M. Moore, Brenna C. Novotny, Peiliang Lou, Ruifeng Guo, Zachary C. Fogarty, Yuanhang Liu, Hongfang Liu, Ellen L. Goode, Stacey J. Winham, Svetomir N. Markovic, Chen Wang

**Affiliations:** 1. Department of Quantitative Health Sciences, Mayo Clinic, Rochester MN. USA; 2. Department of Oncology, Mayo Clinic, Rochester MN. USA; 3. Department of Laboratory Medicine and Pathology, Mayo Clinic, Jacksonville, FL. USA; 4. McWilliams School of Biomedical Informatics, The University of Texas Health Science Center at Houston, Houston, TX. USA

**Keywords:** TMA core registration, Cell feature validation, Multiplex immunofluorescence interpretation

## Abstract

**Background:**

Multiplexed immunofluorescence (MxIF) enables high-dimensional immune cell phenotyping and detailed characterization of the tumor immune microenvironment (TIME), but complex cyclic staining can introduce signal degradation and uncertainty in cell-level measurements. In routine practice, pathologists rely on hematoxylin and eosin (H&E) staining as the primary reference for tissue morphology and frequently cross-reference H&E to interpret MxIF findings.

**Methods:**

We present a framework for aligning H&E and MxIF images at the tissue microarray (TMA) core level to support cross-modal analysis. Cell nucleus detections from each modality are used as anchor points, formulating the task as a point-set alignment problem. Coherent Point Drift (CPD) is applied for global alignment, followed by graph-matching–based refinement to improve local consistency. Evaluation on ovarian TMAs demonstrates consistent alignment performance for both restained and serial sections. We further explore the use of restained H&E as a reference for MxIF interpretation and investigate virtual H&E generation from MxIF data as a complementary approach when restained H&E is unavailable.

**Results:**

The aligned images enable quantitative assessment of cross-modal feature concordance between MxIF-derived measurements and H&E morphology. The generated virtual H&E demonstrated similar cell population compared to the real H&E.

**Conclusions:**

Cell-centric alignment can facilitate integrative multimodal histopathology analysis for TMA cores in spatial co-localization, cell feature validation, and virtual H&E generation.

## Introduction:

As an important approach to reveal cell level details in cancer, histopathological images have been widely used in both clinical practice for diagnostic decision making and treatment follow up. Following different staining protocols, each modality of histopathology has its unique strength in highlighting specific aspects within the tumor immune microenvironment (TIME). Among them, multiplexed Immunofluorescence (MxIF) images provide refined immune cell phenotyping, making it a favorable research tool for revealing cell behaviors in TIME. However, MxIF has yet to see widespread adoption in clinical practice, partially due to challenges in maintaining marker signal integrity during the complex cyclic staining processes. On the other hand, H&E (Hematoxylin and Eosin) staining plays an irreplaceable role in providing standard clinical references by revealing cell morphology and texture patterns (Supplementary Figure 1). Thus, accumulating studies have been combining cell level information from H&E with other histopathology stains to interrogate the mechanism of cancer development and metastasis. For example, Bao etc. proposed a bifocal neural network to explicitly learn from H&E and IHC (Immunohistochemistry) for identifying abnormalities in pathology images [1]. Gatenbee etc. [2, 3] compared the characterization of TIME using MxIF and H&E. Overall, the prerequisite of integrative multimodal histopathology TIME analysis is to approximate cell-level spatial correspondence between multimodalities, as the combined cell level characterization relies on co-localized cells within cancer tissues.

Although it is feasible to align diverse histopathological staining by annotating a few landmarks, this labor-intensive labeling work makes it hard to be applied to all the images in a large cohort study, especially for tissue micro-arrays (TMAs), as each whole slide image (WSI) contains hundreds of tissue cores. Image registration is an automatic technique that has been widely used for medical image alignment to establish pixel level correspondence. For example. Jonsson etc. [4] developed an image registration method targeted towards computer-aided voxel-wise analysis of whole-body PET-CT data. Jiang etc. developed an automatic alignment method by capturing the hierarchical nature of whole slide images [5]. Although image registration has been leveraged in histopathology images, it is still challenging to achieve cell-level alignment for the following reasons. First, the size of histopathology images (usually more than 3GB) is much larger than other medical images, such as CT or MR (usually less than 1MB), which means many existing methods can’t be directly applied in our scenario due to the limitation of computational resource. Second, the contents (cells) within histopathology imaging are dense and small compared to the image size, which exhausts the algorithm to converge; Third, the content of images acquired from diverse modalities can be dramatically different. Some cells in one modality may not match the compartments in another modality even if the images are from the restained tissue section, as the pigment only bonds to cells with specific markers. In consequence, algorithms may not be able to find enough reliable landmarks to establish the location correspondence. Thus, there exists a practical need for multimodal histopathology alignment to enable integrating detailed information from different stains.

Given the challenges of image alignment by directly using pixel information, it is straightforward to consider using cell segmentation outcomes from histopathology images as the starting point to develop approaches for establishing cell spatial correspondence. This resolving path does not incur extra cost to the existing workflow since cell segmentation is the premise of cell level downstream spatial analysis. On the contrary, it can significantly reduce computational complexity as the number of cells is dramatically less than the number of pixels within a histopathology image. Along this resolving path, two major components are required: 1) cell segmentation; 2) point set alignment. Cell segmentation denotes the methods to detect cells and locate the boundaries of cells from histopathological images. With the advancement of AI and Deep Learning, many cell segmentation models achieve not only superior performance but also high generalizability and robustness[6–8]. Since cell segmentation is comparable to the key points detection steps within the traditional image registration process, the advancement in AI-enhanced cell segmentation can be fully leveraged in this solving path. With the centroids of cell segmentations, cells within histopathology images can be summarized as a point set. Thus, establishing cell spatial correspondence can be formulated as a point sets alignment problem, which has also been widely explored within the machine learning and pattern recognition field [9, 10].

**Figure 1.**
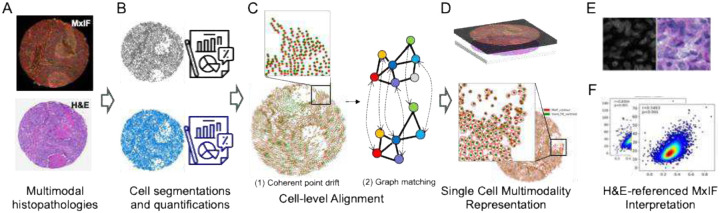
Overview of the framework. A) MxIF and H&E core images to be aligned; B) Cell segmentations and quantifications; C) Cell-level alignment. 1) coherent point drift. 2) graph matching; D) Aligned MxIF and H&E images with combined cell-level representations. E) Virtual H&E generation and F) Cell feature validation enhanced H&E-referenced MxIF interpretation.

In this paper, we present a framework to establish spatial correspondence between cells from different histopathology stainings, allowing us to validate cell features across different modalities and enabling the synergy of combinational analysis (Fig. 1). By formulating the cell spatial correspondence into point sets alignment, we used cell segmentation results from histopathology image pairs as the basis, cells were first aligned with Coherent Point Drift (CPD) [10] and then the alignment was calibrated using Graph Matching (GM)[11]. We systematically evaluated the alignment accuracy for both restained and serial section images from ovarian cancer TMA images. The proposed method achieves encouraging alignment accuracy and is portable to different cell segmentation methods. Within the aligned multimodal image pairs, we found that cell morphology and nucleus staining features are highly correlated, especially in restained tissue cores. Regional analysis also confirmed high concordance in cell density and cell composition despite discrepancies in automatic cell segmentation results within different modalities. Moreover, the multimodality alignment enabled training a deep learning model which generates high fidelity virtual H&E, providing clinical reference for MxIF images.

## Methods:

### 1. Dataset and materials

#### Slide preparation, image acquisition and preprocessing.

The dataset used for this study consists of three ovarian tissue micro array (TMA) images (whole slide scanning) from two tissue sections (Supplementary Figure 2). To create this TMA, ovarian tissues were retrieved from the Mayo Clinic Ovarian Tissue Archive, punched into arrays and made into formalin-fixed paraffin-embedded (FFPE) blocks. Two continuous 5μm sections were cut for MxIF and H&E staining.

For the first section (Sec1 in Supplementary Figure 2), a sophisticated approach involving cyclic MxIF (Multiplexed Immunofluorescence) staining was employed to meticulously assess protein expression levels within individual cells. This method enabled iterative quantification, wherein tissues were sequentially subjected to fluorescent antibody staining, digitally scanned, and then the dye was inactivated to facilitate successive rounds of staining and imaging. The raw MxIF images from CellDive were processed using our previous preprocessing pipeline to remove auto-fluorescence (AF) and convert into OME TIFF files[12]. Following the completion of MxIF imaging, the restained section was restained with H&E (Hematoxylin and Eosin), providing additional morphological context to complement the molecular insights garnered through MxIF. The second section (Sec2 in Supplementary Figure 2) only underwent H&E staining. Specifically, the MxIF images were obtained from CellDive, and H&E images were from Zeiss Axio Z1. Images from both sections were used in our method development and evaluations.

Since the TMA images consist of many FOVs (fields of view) and each FOV is a tissue core image, the TMA images were de-arrayed within QuPath [13] for downstream alignment and evaluation. An ID was assigned to each tissue core. The extracted TMA core images were visually inspected and the tissue cores without sufficient tissues were discarded. In total, three TMA whole slide images (WSIs) were processed, 358 tissue cores images were extracted from each WSI.

StarDist (QuPath implementations) was used as our baseline cell segmentation[14]. Pretrained StarDist models ('he_heavy_augmen' and 'dsb2018_heavy_augmen', refer to our code for more details) for H&E and MxIF were applied to our dataset to get cell segmentations which were then imported into QuPath (version 0.5.0) for quantification. To investigate how the cell segmentation results affect the alignment, we also included a QuPath’s built-in cell detection algorithm watershed for comparison.

### 2. Alignment Algorithms

#### A. Problem Formulation

Considering the scenarios in histopathology image scanning, three transformations were included in for alignment, in which scaling results from the differences of pixel spacing within two images, rotation and translation result from the differences in the tissue locations. The overall transformation from one image to another can be summarized into a transformation matrix *M*.


(1) $$M=\left[\begin{array}{ccc} S*\text{cos}\left(\theta \right) & -\text{sin}\left(\theta \right) & dx\\ \text{sin}\left(\theta \right) & S*\text{cos}\left(\theta \right) & dy\\ 0 & 0 & 1 \end{array}\right]$$

In this formular, the scaling was denoted by *S*, rotation was denoted by *θ*. The translation/shifting distances on *x* and *y* directions were denoted by *dx* and *dy*, respectively. The distance of translation *T* can be calculated as $\sqrt{dx^{2}+dy^{2}}$.

### B. Coherent Point Drift (CPD)

**Figure 2.**
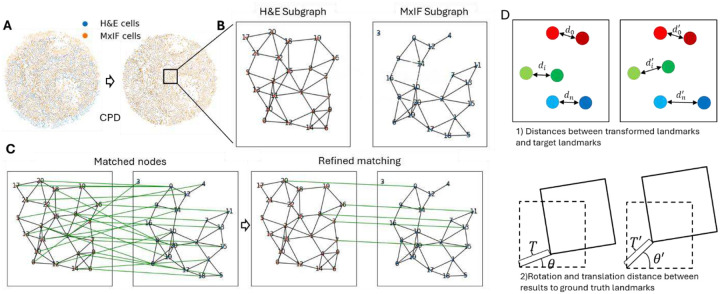
Illustration of alignment algorithms and alignment accuracy evaluation method. (A) Cell centroids were first aligned with CPD, and then fine-tuned with GM. Within the GM phase, (B) subgraphs were created for H&E and MxIF subregions with raw aligned cells as graph nodes. (C) The putative matching nodes were then filtered to establish the correct cell correspondence. (D) Alignment was evaluated with 1) Distances between landmarks after transformation (red dots) and target landmarks (green dots). 2) Rotation and translation. Landmark distances, translations and rotations were denoted by *d*_*i*_,*T* *and* *θ* in ground truth and $d_{i}^{\prime }$, *θ*′ *and* *T*′ within our results.

CPD was employed to generate a raw alignment for cell centroids (Fig. 2A). This method formulates the point-set registration as a probability density estimation problem, where one point cloud is represented using a Gaussian Mixture Model (GMM). Considering the transformation defined in formula (1), the original CPD algorithm needs to update three major parameters (*θ,S and T)* for the alignment. As an Estimation-Maximization (EM) method, CPD iteratively estimates the point correspondence and updates the three parameters until convergence [10].

Specifically, to reduce the degrees of freedom within the CPD algorithm, the cell centroid coordinates were converted into micrometers (μm), so the scale value within formula (1) can be set to a constant value (*S*= 1). In our case, the pixel size of MxIF and H&E image are 0.325μm and 0.212μm, respectively. Pixel coordinates can be converted to micron units by multiplying by the pixel size.

### C. Graph Matching (GM)

Since the CPD-based method estimates the point correspondence using GMM, the alignment was estimated based on the cell densities rather than one-to-one correspondence. To calibrate the alignment, we formulated the cell-to-cell correspondence as a graph matching problem, which was implemented as a module concatenated to the CPD in our framework (Fig. 1C). In this phase, the cells from H&E or MxIF were abstracted as nodes in a mathematical graph.

#### C1. Creating subgraphs

After the CPD transformation, the cells from H&E and MxIF were roughly aligned. Two subgraphs, the source graph from H&E and the target graph from MxIF were created for matching. Since the subregions with denser cell counts usually present significant tissue architectures, while the regions with sparse cells can more likely be affected by cell segmentation noise, the cells for graph creation were randomly sampled from subregions with dense cell distributions. Kernel density estimation (KDE) was used to calculate density value d for each cell; cells with d>=0.5 were optional cells for sampling. To create a source graph for H&E, the cells were sampled from a window with size of 50 μm (Fig. 2B). For the target graph in MxIF, the location of the sampling window was calculated by mapping the centroid of sampling window in H&E using CPD transformation, and the window size was set to 150 μm. The edges for both source and target graphs were added based on proximity of cells if the pairwise distance was less than 15 μm.

#### C2. Node matching

Graph matching is a computational technique that seeks to establish node-to-node correspondence between two or more graphs. This process involves solving a combinatorial optimization problem, which is typically NP-hard. The goal is to match nodes from one graph to another while considering the similarity of the nodes (node affinity) and the relationships between connected nodes (edge affinity). By considering both types of affinities, graph matching methods tend to be more resilient to noise and outliers in the data, making them suitable for complex real-world applications.

In our case, the affinity matrices were built by calculating node and edge similarities between corresponding graphs. Node features, such as cell morphologies (perimeter, solidity), were used to quantify node affinity. Edge affinity was based on the distances between connected nodes, with edge features capturing the structural relationship between node pairs in the graph. A Reweighted Random Walks Matching (RRWM) solver was then used to iteratively refine the matching score based on the affinity matrix, which yields a probability distribution for potential matches. These soft matching results were converted into a final “hard” matching using a combinatorial optimization method like the Hungarian algorithm or the Sinkhorn algorithm[11, 15].

#### C3. Filtering matched node pairs

Although graph matching establishes the one-to-one correspondence between nodes in source and target graphs, not all the node correspondences are true positive. Considering the cells segmented out of the original H&E and MxIF images also represented the microstructures of ovarian tissue images, we introduced Locality Preserving Matching (LPM)[16] to exclude unreliable matching node pairs for the final spatial correspondence, as this method was explicitly designed to filter putative matching point pairs while preserving the spatial neighborhood relationship among points. Fig. 2C illustrates the graph matching results before and after LPM filtering, only the matching pairs preserving the neighborhood relationships were kept. In our case, the graph nodes in the source plot were the cell centroids from H&E segmentation, while the nodes in the target plot were cell centroids from MxIF. After the matching point pairs filtering, the left point pairs were used to calculate the affine transformation that translocates H&E cell locations to MxIF cell locations. To implement our framework, three major python packages StarDist [14], probreg [17, 18] and pygmtools [11] were used. More details about the implementations can be found in our shared GitHub code (https://github.com/dimi-lab/MultimodalityHistoComb).

### 3. Alignment Accuracy Evaluation

#### A. Creating alignment ground truth

The ground truth of cell level correspondence was established by annotating landmarks within H&E and MxIF image pairs from 20 TMA cores without significant tissue damage. For each selected pair of tissue cores, MxIF and H&E images were imported into QuPath, in which the point annotation tool was used to annotate eight pairs of landmarks (Supplementary Fig. 3). To efficiently locate corresponding cell pairs within H&E and MxIF, significant microarchitectures, such as interface of tumor and stroma, were visually referenced to navigate to individual cells. Landmark points were placed at the center of the nucleus close to significant microarchitectures in both H&E and MxIF. To minimize annotation errors, lymphocytes were preferred as landmarks, since their nuclei are dark and small. Then the coordinates of annotated landmarks were exported from QuPath to calculate the ground truth transformation matrix M, in which the rotation *θ*, scale *S* and translation *dx* and *dy* for the rigid alignment were summarized in formula (1).

#### B. Evaluation metrics

To quantitatively evaluate the performance of alignment accuracy, three metrics were used by referring to existing work [2], as summarized in formula (2). We compared the differences between alignment results from annotation and our methods. Following the completion of alignment, the location of landmarks annotated in the source image were mapped to the new locations within the target image, labeled with red dots in Fig. 2D. The average distance between landmarks in target image (green dots) and transformed landmarks from source image (red dots) was used to measure the overall alignment accuracy, denoted by Δ*D* in (2). With the annotated landmarks, the ground truth rotation angle *θ* and translation distance *T* can be calculated. The difference between the results from our method and the ground truth were the other two quantitative metrics, denoted by Δ*θ* and Δ*T* respectively. Using the defined evaluation metrics, alignment accuracies were evaluated for re-stained and serial sections.


(2) $$\Delta D=\frac{1}{n}\sum_{i=0}^{n}\left|d_{i}-d_{i}^{\prime }\right|\;\Delta T=\left|T^{\prime }-T\right|\;\Delta =\left|\theta^{\prime }-\theta \right|$$

### 4. Downstream Analysis

To interpret dark field MxIF images effectively, H&E-stained counterparts are commonly used to provide cell-level referencing. Ideally, the same tissue section used for MxIF should be restained with H&E after the MxIF scanning to ensure identical referencing. However, the tissue may become damaged after multiple rounds of MxIF imaging, resulting in incomplete or compromised H&E staining. Consequently, many MxIF platforms offer virtual H&E images, though the quality is often insufficient for clinical purposes. Another common approach is to use serial sections from the same tissue block for H&E staining. However, these serial sections provide a less reliable reference compared to restained H&E, as the cells do not always correspond one-to-one with those in the original MxIF images. This raises two important questions: 1) How reliable are serial section H&E images as references for MxIF analysis when restained H&E is not available? 2) Can high-quality virtual H&E images be generated from MxIF data to serve as a reliable reference, similar to restained H&E?

Using our specific ovarian TMA dataset, we demonstrated that our alignment method addresses these challenges by 1) validating cell features across MxIF and H&E (both restained and serial), and 2) facilitating the training of a deep learning model that generates high-fidelity virtual H&E images directly from MxIF image channels. This approach provides a practical solution for reliable referencing in MxIF analysis without the need for additional tissue sectioning and staining.

#### A. Cell feature concordance

Pathologists rely on tissue staining and cell morphology to make accurate diagnoses, so automating the extraction of cell features is crucial for replicating their interpretative processes, especially in large-scale studies. By analyzing the colocalization of cell nucleus staining and morphological characteristics, we can evaluate the consistency of features at both the cellular and regional levels. This ensures the quality of MxIF data and the reliability of downstream analyses.

To achieve this, cells were identified using segmentation models [7], and quantitative cellular features were extracted for both MxIF and H&E images using QuPath[19]. These features included measurements such as average DAPI or Hematoxylin signal intensity per cell and the area of the cell nucleus. Comparisons of these measurements were made either at the individual cell level or across regions for both restained and serial tissue sections. This process is critical for ensuring that cell-based analyses, including those related to tissue architecture and pathology, are robust and interpretable. Additionally, such automated feature extraction supports the scalability of pathology workflows by providing standardized, objective measurements for large cohort studies.

#### B. Generating virtual H&E from MxIF

Using our alignment method and dataset, we demonstrated the feasibility of generating high-fidelity virtual H&E images by training a deep generative model on selected image channels from existing MxIF data. The architecture of the deep learning model was presented in our previous work[20]. Since DAPI (4′,6-diamidino-2-phenylindole), NAK (Na/K-ATPase) and PANCK (pan-cytokeratin) are three important markers for nucleus and membrane staining, these three channels were extracted from MxIF as one of the inputs (condition) for training the conditional Generative Adversarial Network (cGAN). In the training phase, the generated image (virtual H&E) from the generator of cGAN was compared with the real H&E (Fig. 6A). In the testing phase, the qualities of generated virtual H&E images were both quantitatively evaluated using quality metrics and visually inspected by pathologists. Using HoverNet [21] as the automatic cell classification model, downstream cell composition was also analyzed to evaluate the consistency of cell population between the real and virtual H&E images.

## Results:

### 1. Our framework achieves approximate cell-level alignment for MxIF and H&E images.

**Figure 3.**
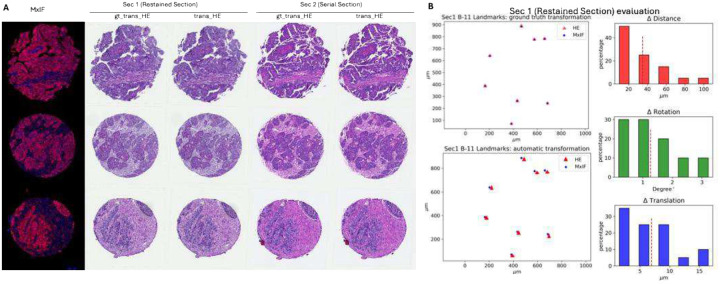
Qualitative and quantitative evaluation results. A) Examples of MxIF and aligned H&E for both restained and serial tissue sections. B) A restained section example of aligned landmarks obtained from annotation (ground truth) and our (automatic) method. The right side is the quantitative evaluation metrics in the all the annotated restained cases.

Our method was tested on both restained and serial sections. For each section and each core, the H&E images were aligned to MxIF. According to the landmark annotations, ground truth transformation *M* was calculated and applied to the restained and serial section. The ground truth results H&E are visualized in Fig. 3A column 2 and 4, while the results from our method are visualized in column 3 and 5. For both sections, our H&E results showed good visual agreement to the corresponding ground truth H&E, even for the broken-up tissue core, shown in the first row of Fig. 3A. Meanwhile, our H&E results for both sections showed good spatial agreement with MxIF, even though there were micro architecture and staining color differences between the two sections.

To visually check the alignment accuracy for the restained sections, the landmarks from H&E (red dots) were translocated according to manually annotation and our automatic method respectively and then plotted together with landmarks from MxIF (blue dots). As an example (tissue core ID: B-11) shown in Fig. 3B, the MxIF landmarks were fully overlayed on translocated H&E landmarks, indicating that for the restained section and manual annotation, the alignment error was minor. The scatter plot also indicated that the automatic alignment errors were higher than the ground truth alignment. According to the definition of our evaluation (formular (2)), the histograms of the quantitative metrics were plotted in Fig. 3B, with Δ*D*_sec1_ = 32.47 ± 27.25, Δ*T* = 6.94 ± 4.73 and Δ*θ* = 1.26 ± 0.98. Average values were labeled with red dash line. Given typical cell diameters (~10−20 μm), these results indicate alignment at an approximate cellular scale rather than exact one-to-one correspondence.

**Figure 4.**
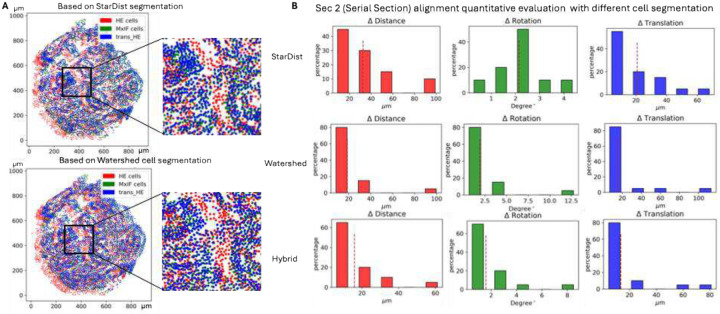
Results of alignment initiated from different cell segmentation methods. A) Cell centroids scatter plot for MxIF (green), original H&E (red) and transformed H&E (blue) from a serial section example. B) Distribution of quantitative metrics for evaluating alignment accuracy using different cell segmentation results as the starting point. First row: StarDist; Second row: Watershed; Third row: StarDist for MxIF while Watershed for H&E.

With the same settings, the alignment accuracy was also evaluated on the serial sections. Results were shown in the first row of Fig. 4B, with Δ*D*_sec2_ = 34.47 ± 25.71,*T* = 20.77 ± 17.46 and Δ*θ* = 2.15 ± 0.97, suggesting that both restained and serial sections can be aligned with reasonable accuracy with manual annotation. However, alignment errors were notably higher for serial sections, particularly in terms of rotation and translation.

To evaluate the generalizability of our alignment method to whole slide images (WSIs), we applied it to a pair of MxIF and H&E images obtained from serial tissue sections. In immunology studies, it is often necessary to identify cellular biomarkers within specific tissue types. While tissue type identification is relatively straightforward in H&E images, it can be challenging in MxIF. Therefore, we first delineated tissue structures on the H&E image and then transferred these annotations to the MxIF image using the MxIF-H&E alignment. These transferred annotations served as spatial references for biomarker identification across distinct tissue types. Given that WSIs contain vastly more cells—often hundreds of times more than a TMA core—direct application of our alignment method is computationally demanding. To address this, we employed a cell sampling strategy to limit the number of points used for Coherent Point Drift (CPD) alignment. Using the resulting transformation, regional annotations from the MxIF image were mapped with consistent spatial correspondence to the corresponding regions in the H&E image (Supplementary Figure 4).

### 2. Our framework is portable to different segmentation models.

Since our method relies on cell segmentation, we investigated the alignment performance with respect to two different segmentation methods, StarDist and Watershed. As the restained tissue section provides more reliable one-to-one cell correspondence, our evaluations were conducted on Section 1. Although Watershed generates more over- and under-segmentation cell instances [22], a similar transformed H&E image can be obtained by applying the transformation from our method. Taking the same tissue core (ID: B-11) as an example, the discrepancies between aligned H&E results images can only be observed by zooming into the cell level and visualizing the cell centroids before and after transformation (Fig. 4A). Moreover, according to the quantitative evaluation metric ΔD, the performance of using Watershed was even better than that using StarDist (Fig. 4B).

We also tested the method using hybrid results from two cell segmentation models, with StarDist for MxIF and Watershed for H&E. Compared to the default segmentation method StarDist, evaluation metric ΔD was lower when using hybrid cell segmentation results for alignment with Δ*D*_*StarDist*_ = 32.47 ± 27.25 *vs*. Δ*D*_*hybrid*_ = 15.87219264 ± 14.65349468. The performance fluctuations shown in Fig. 3B and Fig. 4B may result from the inconsistencies within H&E and MxIF cell segmentation. Since there were many cell segmentation models available specifically for H&E or MxIF, our method provided a straightforward and portable way to align cells using segmentation results as the starting point for alignment.

### 3. Multimodality alignments enable cell and regional feature concordance evaluation.

**Figure 5.**
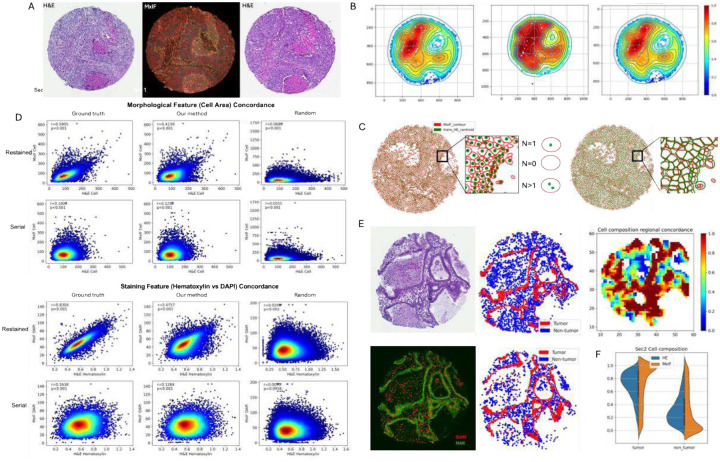
Visualization of aligned cells, cell feature concordance evaluations and cellular composition comparison. A) An example of aligned MxIF and H&E, including both a restained and a serial section. B) Regional cell density for the example. C) An example of aligned cells with cells from MxIF (red) showing cell contours while the cells from H&E(green) show cell centroids (left of the figure). The same example with cells from both H&E (green) and MxIF(red) showing cell contours (right of the figure). D) Cell feature concordance under different conditions. Row 1 and 3: restained section; Row 2 and 4: serial sections; Column 1: aligned with manual annotation; Column 2: aligned with our method; Column 3: randomly align to a tissue core. E) An example of H&E and MxIF. The cells were labeled with red (tumor) and blue (non-tumor). Regional cell composition similarity was illustrated with heatmap (warm color denotes high concordance). F) The comparison of cell composition within H&E and MxIF for all serial tissue cores.

Since our alignment framework aims to enable referenced spatial analysis within the aligned tissue space, cell and regional signal concordances after the alignment were evaluated. By applying the transformation to the cell segmentation results, cells from H&E and MxIF can be aligned to the same space, as shown in Fig. 5A. Although the regional cell densities for MxIF can be higher than that in H&E due to higher cell detection sensitivity, cell densities for H&Es from different sections were almost identical, as shown in Fig. 5B. This fact implies that even if we apply the ground truth transformation, rigid one-to-one cell correspondence cannot always be established for two major reasons: 1) The cells within tissue section images were not identical, especially for serial sections. Ideally, for the restained tissue, the cells should be the same for different imaging techniques. However, there could be cells washed off or even large pieces of tissue damaged during the stain-restain process. 2) The cell segmentation models do not always generate the same segmentation results from different modalities as the models were trained from and applied to different modalities independently. For example, there are pretrained StarDist models available for H&E and MxIF respectively, but the models were trained from different datasets. The results will be different when the model is applied to H&E and MxIF independently.

To check the single-cell-level feature concordance between H&E and MxIF, the restained tissue section was used. By visualizing the aligned cells into the same space, as shown in Fig. 5C, cells from two modalities demonstrated three scenarios in single cell level correspondences: 1) N=1, the cells are one-to-one corresponded; 2) N=0, a cell detected in one image did not correspond to any cell in another image; 3) N>1, more than one cell in one image corresponded to one cell in another. According to our observations, for both N=0 and N>1, the mismatch was caused by segmentation inconsistency between H&E and MxIF. The StarDist model we applied to MxIF was sensitive to DAPI signals, which led to two consequences: 1) For cells with weak DAPI signals, the instances detected in MxIF could be recognized as fragments within H&E which can be filtered out within segmentation process (N=0). 2) For cells close to each other and with strong DAPI signals, multiple instances that are recognizable in H&E can be detected as single cell in MxIF (N>1) (Supplementary Fig. 5). Since N=0 and N>1 were extreme cases for cell segmentation, we investigated the morphological and staining feature concordance with one-to-one cell correspondence (N=1) within all 20 evaluation cores. As shown in Fig. 5D first row, morphological features, such as cell area, from H&E cells and MxIF cells showed moderate correlation. Meanwhile, the staining concordances were compared on DAPI and hematoxylin as both are cell nucleus pigments (Fig. 5D third row). For both morphological and staining features, r and p values for Pearson correlation were calculated and shown in the plots.

To be more generic, we investigated the cell level feature concordance under two different conditions: 1) align with manually labeled landmarks vs. our automatic alignment; 2) serial vs. restained section. Each tissue core was also aligned to a randomly chosen core as the baseline for comparison. Specifically, after alignment, each cell in H&E was corresponded to the nearest MxIF cell for feature comparison. As shown in Fig. 5D second and forth row, both morphological (cell area) and staining features (DAPI vs. Hematoxylin) demonstrated higher feature concordance than the serial section. Though the concordance based on our alignment was lower than the ground truth, it is comparable in both restained and serial section and was significantly higher than random alignment.

Although the cell-level feature concordance in serial sections was lower, similar tissue structures in H&E and MxIF were observed. As shown in Fig. 5E, cells within H&E and MxIF were classified as tumor or non-tumor by referencing the cell features extracted by StarDist models and QuPath[12, 22]. Then, the concordance of cellular composition was evaluated by comparing proportions of tumor cells region-by-region. A heatmap was used to demonstrate the reginal similarity of cell composition, in which warm color indicates high concordance. The most discordant regions significantly overlapped with areas where H&E cell segmentations differed from MxIF cell segmentations. Cell composition differences were also compared for all serial tissue cores. The results indicated that the cell population share similar distribution but more cells were detected within MxIF images (Fig. 5F).

### 4. Alignment enhanced clinical referencing through generating visually realistic virtual H&E

**Figure 6.**
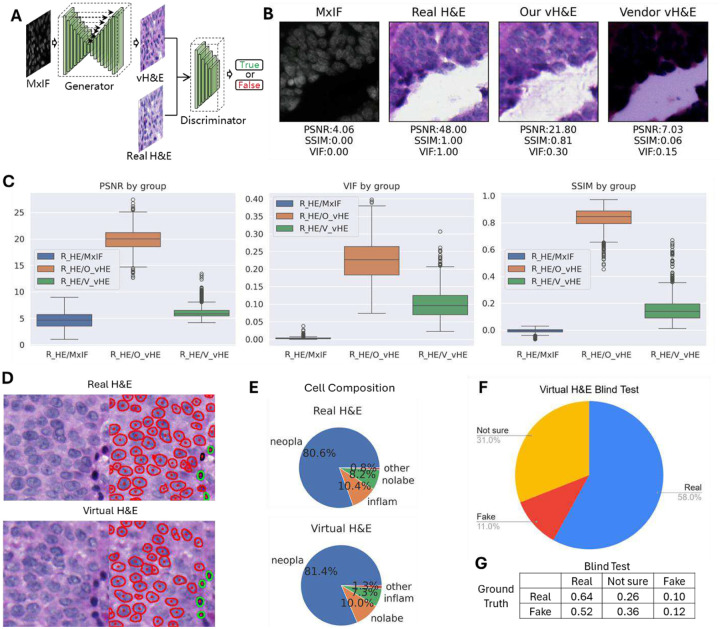
Generating virtual H&E from MxIF and image quality evaluation. A) The schema of the virtual H&E generation model. B) Example image of original MxIF, restained H&E, virtual H&E from our method and virtual H&E from the platform vendor. C) Quantitative evaluation results showing PSNR, VIF and SSIM by different groups. R_HE/MxIF denotes real H&E vs. MxIF, R_HE/O_vHE denotes real H&E vs. our virtual H&E, R_HE/V_vHE denotes real H&E vs. vender’s virtual H&E. D) Example of original real H&E and our virtual H&E images, together with cell detection and classification results overlay. E) Cell composition comparison between real H&E and our virtual H&E on our entire dataset, in which nolabe denotes no label, inflam denotes inflammatory, neopla denotes neoplastic. F) The proportion of “real”, “not sure” and “fake” images in the blind test. G) Comparison between blind test and ground truth.

With aligned real H&E and MxIF image pairs as input (Fig. 6A), the trained cGAN model can generate visually realistic virtual H&E images from DAPI, NAK and PANCK channels within MxIF[20]. The model was trained on 20 aligned TMA cores (5845 patches) and evaluated on a hold-out set of 10 cores (2683 patches). Since the training dataset is small, the model was trained on one GPU server and converged in 28 epochs. According to a pathologist’s visual inspection, the virtual H&E images from our model were visually improved compared to the vendor-provided virtual H&E which was dark and did not allow visualization of cell details (Fig. 6B). Meanwhile, our virtual H&E preserved the tissue context well but generated some light tissue fragments in blank areas which might be the result of the model picking up weak background fluorescence signal from MxIF images. Three evaluation metrics, including PSNR (Peak Signal-to-Noise Ratio) [23], VIF (Visual Information Fidelity) [24] and SSIM (Structural Similarity Index Measure)[25] were also used to intrinsically measure the quality of virtual H&E images by comparing them to the corresponding real H&E[20]. Our boxplots indicated that our virtual H&E achieved the highest similarity to the real H&E (Fig. 6C).

To extrinsically evaluate the quality of generated H&E images, cell compositions within real and our virtual H&E were compared. A pretrained model MoNuSAC [26] derived from HoverNet [21] was used to classify cells in both real and virtual H&E (Fig. 6D). Using the default configuration, the detected cells were classified into four groups: inflammatory, neoplastic, other, and no label. Based on the automatic cell classification, we observed that real H&E and virtual H&E share a similar cell population, with 3.1% difference in inflammatory cells, and less than 1% difference in neoplastic (Fig. 6E). The similarity of cell composition between real H&E and virtual H&E suggested that the generated images may serve as a complementary reference for interpretation.

A blind evaluation was designed to validate the fidelity of the virtual H&E from our method. 200 images (50% real H&E and 50% our virtual H&E) were randomly selected from our dataset and loaded into a Google Form. Without knowing the proportion of real images, pathologists were invited to label each image to “real”, “not sure” or “fake” according to their impression to the image. The summarization of this blind test indicates that 58% of images were considered to be “real”, though only 50% are from real H&E (Fig. 6F). Further investigation found that 52% of our virtual H&E passed the test. Based on the pathologist manual review, in some cases it is difficult to distinguish virtual H&Es from real to fake especially for the ones with considerable cellular compositions as well as acceptable resolutions. For both real and virtual H&E, many of uncertainties arose from images that were blur/out of focus, as well as those with low cellularity, increased stromal content, or other non-cellular components.

## Discussions

We introduced a novel histopathology image registration framework that relies solely on cell segmentation results, which are prerequisites for many downstream analyses. This approach allows for the integration of cell features from multiple histopathology modalities without requiring significant modifications to existing analysis pipelines. The method demonstrated stable performance under the evaluated conditions, as the convergence of the CPD (Coherent Point Drift) algorithm primarily depends on regional cell densities. Furthermore, we demonstrated the portability of our approach across different segmentation techniques. Although the graph matching step operates under strict conditions, it opens the door to incorporating advanced techniques, such as graph neural networks, to measure the similarity between nodes and approximate cell-level spatial correspondence. Developed and evaluated on a specific dataset, our work not only showed promising alignment accuracy but also highlighted potential pitfalls that can arise under various tissue cutting and staining conditions.

While existing approaches often treat histopathology image alignment as a non-rigid registration problem due to tissue warping [27], we simplified TMA (tissue microarray) image alignment as a rigid transformation. There are two main reasons for this: first, compared to whole-slide images, TMA core images are much smaller (WSI: ~40,000 pixels in height vs. TMA core: ~4000 pixels in height). Based on our observations, significant tissue warping is more prevalent in bulk tissue whole slide images than in TMA cores. Second, tissue warping should ideally be avoided during slide preparation to ensure reliable downstream analysis, as non-rigid registration can only adjust for the warping rather than eliminate alignment errors. In fact, the discontinuous control points used in non-rigid registration methods can cause regional distortions [28].

Our alignment method was thoroughly evaluated on both serial and restained sections of TMA slides. This is particularly valuable for TMA slides, as TMA scanning is a more practical imaging approach for large cohort studies [30]. Manually aligning each tissue core within a TMA slide is nearly infeasible due to the sheer volume and complexity. In contrast, achieving rigid image registration in whole-slide images requires relatively low annotation effort—only two landmarks per image pair are sufficient to estimate the transformation in Equation (1). Although our method has been tested on WSIs and has demonstrated its potential for transferring annotations from MxIF to H&E images, further comprehensive evaluations are needed to validate its robustness and accuracy across larger datasets. Additionally, WSIs are more susceptible to regional distortions and tissue folding, which significantly limits the effectiveness of rigid alignment. While some existing studies have employed rigid and non-rigid registration techniques to align image patches or regions, it remains challenging to seamlessly reconstruct a coherent whole-slide image, even with local alignment correction and patch fusion.

A key limitation of this study is the relatively small sample size (three whole slide images, each with 358 tissue cores) and the use of TMA sections from a single ovarian cohort, which may limit the generalizability of the findings. However, the dataset is uniquely curated to include paired H&E and MxIF images with cell-level correspondence, enabling detailed cross-modal validation that is not feasible with conventional large-scale datasets. As such, the study prioritizes methodological validation and downstream analytical capability over large-scale benchmarking. Future work will focus on extending the proposed framework to larger and more diverse datasets as they become available.

In our comparative analysis, we applied the same evaluation procedure to two baseline methods traditional SIFT [29] and STalign [30]. As a well-established method, SIFT-based alignment demonstrated strong performance, particularly in minimizing landmark distance and rotation angle. However, its translation error was comparable to that of our method, likely due to occasional failure in detecting reliable key points prior to affine transformation. Additionally, SIFT was unable to align whole slide images because the image size exceeded its processing limits. STalign, on the other hand, is a recently developed method designed for aligning spatial omics data. Conceptually, it is capable of handling whole slide images and compensating for tissue distortion. However, in our experiments, it posed practical challenges, particularly in parameter tuning, and ultimately produced inferior alignment results. (Supplementary Figure 6) These comparisons highlight the inherent difficulty of multimodal histopathology alignment and underscore the need for selecting methods that are well-suited to specific application scenarios. Despite some variability in alignment accuracy across methods, all consistently outperformed when applied to restained tissue sections, achieving notably higher alignment precision than in the serial sections.

For the cell feature concordance evaluation, our work provided significant insights to H&E referenced MxIF interpretation by aligning both restained and serial H&E section to MxIF. At the cellular level, cell morphology features were found to be more reliable in H&E images based on pathologists' observations. In our dataset, some cells identified in MxIF appeared as fragments in the H&E images, while others that seemed to be a single cell in MxIF were actually two or three distinct cells in H&E, likely due to strong fluorescence signals spilling over into adjacent cells. Furthermore, our evaluation of cell feature concordance revealed that the agreement between restained H&E and MxIF was much higher than between serial H&E and MxIF, suggesting that restained H&E is preferable for cell-level referencing in MxIF analysis. At the regional level, we observed similar cell densities between restained and serial H&E, and both showed comparable regional cell composition with MxIF, albeit with some overall differences. Discrepancies in cell segmentation and tissue classification introduced uncertainty in combined downstream analysis, but also provided an opportunity to cross-validate image quality once the images were aligned.

Our evaluations at both the cellular and regional levels suggested that restained H&E offers a more reliable reference for MxIF analysis. Consequently, we explored the possibility of generating high-quality virtual H&E images from MxIF data. This approach not only eliminates the need for additional tissue sectioning, staining, and scanning but also preserves cell-level similarities, making it suitable for referenced MxIF study. Although training a GAN-based model is notoriously challenging, we encountered relatively few difficulties during the training phase, indicating that our method could potentially be generalized to other tissue types, provided suitable datasets are available for fine-tuning.

Upon closely inspecting the model outputs, some defects were observed: 1) The model occasionally generated artifacts in tissue regions, which is a common behavior in generative models and could potentially be used to identify image quality issues within certain MxIF markers. 2) While the color of the virtual H&E images closely resembled that of real H&E, the cell details were generally less sharp, which may contribute to lower accuracy in automated cell segmentation and classification compared to real H&E. Overall, while the virtual H&E images from our method were not as impeccable as the restained H&E, they provided useful MxIF referencing with substantially higher quality than the default virtual H&E from the platform.

Our work creates new opportunities for learning latent information from multiple modalities of histopathology images in an integrated way. Within co-localized histopathology images, we can utilize complementary information from each modality to enrich cell-level and regional representations. The success of models like CLIP [31] for text-image learning inspired us to explore the possibility of applying similar approaches for jointly learning from multiple histopathology modalities. However, the design of current text-image models may not be ideally suited for the medical domain, where clinical notes generally offer an overall summary of a patien’s condition, while histopathology image tiles capture highly localized information. This disparity in data granularity poses a challenge for existing text-image alignment methods, which are limited by these global versus local perspectives. In contrast, when dealing solely with image data, aligning different modalities resolves the granularity gap, allowing for more refined co-training approaches, such as patch-to-patch or even cell-to-cell alignment. Our method thus provides a basis for future exploration of more precise multi-modal learning, enabling new downstream applications that can fully exploit the complementary nature of different histopathology imaging modalities.

## Conclusion

This study presents a cell-centric framework for aligning H&E and MxIF images at the TMA core level. The results demonstrate that the proposed approach can achieve consistent spatial alignment under the evaluated conditions and enables downstream analyses such as cross-modal feature comparison and virtual H&E generation. While the study is limited by dataset size and scope, it provides a practical foundation for future work in multimodal histopathology integration.

## Supplementary Material

This is a list of supplementary files associated with this preprint. Click to download.

- SupplementaryFigures0529.docx

Supplementary Figure 1: Importance of H&E Alignment with MxIF for Quality Control in Spatial-Omics Data, including A) Positive, B) Negative and C) Mixed examples.

Supplementary Figure 2. Ovarian TMA dataset used in this study. A) and B) were a tissue section (Section 1) stained with MxIF protocol and then restained with H&E. C) was H&E stain of a serial section (Section 2) 5 μm apart from section 1.

Supplementary Figure 3. TMA core with manually labeled landmarks (eight points annotations). Each yellow dot was a landmark manually label for establishing ground truth transformation. The size of the landmarks was enlarged for better visualization. The actual resolution of landmarks equals to the pixel size of the image.

Supplementary Figure 4. A) Example of cell sampling results from a pair of aligned images: MxIF (top) and H&E (bottom). B) Visualization of alignment results. Tissue structures were delineated on the H&E image and subsequently transferred to the MxIF image based-on the MxIF-H&E alignment. These transferred annotations serve as references for identifying biomarkers across different tissue types.

Supplementary Figure 5. Cell morphology differences caused by segmentation discrepancies. A) concordance of cell morphology features; B) segmentation differences within MxIF and H&E.

Supplementary Figure 6. Alignment accuracy comparison on restained and serial TMA sections. A) SIFT; B) STalign.

## Data Availability

The dataset used in this study is publicly available at https://immunoatlas.org/MYCB/240802-1/MYCB24004/Code, example data and documentation for the proposed framework are accessible via GitHub: https://github.com/dimi-lab/MultimodalityHistoComb Details of the virtual vs. real H&E blind test can be found in this Google Form: https://docs.google.com/forms/d/e/1FAIpQLScAYf_t8eBe2Oo6ac3SWS6i9QstyUOVQqT1GEobic05gmlhKA/viewform?vc=0&c=0&w=1&flr=0

## References

[1] Bao G., Graeber M.B., and Wang X.. A bifocal classification and fusion network for multimodal image analysis in histopathology. in 2020 16th International Conference on Control, Automation, Robotics and Vision (ICARCV). 2020. IEEE.

[2] Gatenbee C.D., , Virtual alignment of pathology image series for multi-gigapixel whole slide images. Nature communications, 2023. 14(1): p. 4502. 10.1038/s41467-023-40218-9 PMC10372014 37495577

[3] Gootkind F.D., , 1287 Comparison of multiplex immunofluorescence and H&E-based approaches for characterization of the tumor microenvironment. 2023, BMJ Specialist Journals.

[4] Jönsson H., , An image registration method for voxel-wise analysis of whole-body oncological PET-CT. Scientific Reports, 2022. 12(1): p. 18768. 36335130 10.1038/s41598-022-23361-z PMC9637131

[5] Jiang J., , Robust Hierarchical Density Estimation and Regression for Re-stained Histological Whole Slide Image Co-registration. BioRxiv, 2019: p. 565564. 10.1371/journal.pone.0220074 PMC6655785 31339943

[6] Bannon D., , DeepCell Kiosk: scaling deep learning–enabled cellular image analysis with Kubernetes. Nature methods, 2021. 18(1): p. 43–45. 33398191 10.1038/s41592-020-01023-0 PMC8759612

[7] Stevens M., , StarDist image segmentation improves circulating tumor cell detection. Cancers, 2022. 14(12): p. 2916. 35740582 10.3390/cancers14122916 PMC9221404

[8] Stringer C., , Cellpose: a generalist algorithm for cellular segmentation. Nature methods, 2021. 18(1): p. 100–106. 33318659 10.1038/s41592-020-01018-x

[9] Feng X.-W., Feng D.-Z., and Zhu Y., Fast Coherent Point Drift. arXiv preprint arXiv:2006.06281, 2020.

[10] Myronenko A. and Song X., Point set registration: Coherent point drift. IEEE transactions on pattern analysis and machine intelligence, 2010. 32(12): p. 2262–2275. 20975122 10.1109/TPAMI.2010.46

[11] Wang R., , Pygmtools: A python graph matching toolkit. Journal of Machine Learning Research, 2024. 25: p. 1–7.41334350

[12] Maus R.L., , Resolving the heterogeneous tumor-centric cellular neighborhood through multiplexed, spatial paracrine interactions in the setting of immune checkpoint blockade. Cancer research communications, 2022. 2(2): p. 78–89. 35992329 10.1158/2767-9764.CRC-21-0146 PMC9390837

[13] Bankhead P., , QuPath: Open source software for digital pathology image analysis. Scientific reports, 2017. 7(1): p. 1–7. 29203879 10.1038/s41598-017-17204-5 PMC5715110

[14] Schmidt U., Cell detection with star-convex polygons. in Medical image computing and computer assisted intervention–MICCAI 2018: 21st international conference, Granada, Spain, September 16–20, 2018, proceedings, part II 11. 2018. Springer.

[15] Ling X., , Multilevel graph matching networks for deep graph similarity learning. IEEE Transactions on Neural Networks and Learning Systems, 2021. 34(2): p. 799–813. 10.1109/TNNLS.2021.3102234 34406948

[16] Ali S., , Ink removal from histopathology whole slide images by combining classification, detection and image generation models. arXiv preprint arXiv:1905.04385, 2019.

[17] Krüger J., A probabilistic approach for the registration of images with missing correspondences. in Medical Imaging 2019: Image Processing. 2019. SPIE.

[18] Kenta T. and , probreg. 2019.

[19] Bankhead P., , QuPath: Open source software for digital pathology image analysis. Scientific reports, 2017. 7(1): p. 16878. 29203879 10.1038/s41598-017-17204-5 PMC5715110

[20] Jiang J., , Image-to-image translation for automatic ink removal in whole slide images. Journal of Medical Imaging, 2020. 7(5): p. 057502–057502. 33102624 10.1117/1.JMI.7.5.057502 PMC7567133

[21] Graham S., , Hover-net: Simultaneous segmentation and classification of nuclei in multi-tissue histology images. Medical image analysis, 2019. 58: p. 101563. 31561183 10.1016/j.media.2019.101563

[22] Jiang J., , Digital pathology-based study of cell-and tissue-level morphologic features in serous borderline ovarian tumor and high-grade serous ovarian cancer. Journal of Pathology Informatics, 2021. 12(1): p. 24. 34447604 10.4103/jpi.jpi_76_20 PMC8356706

[23] Korhonen J. and You J.. Peak signal-to-noise ratio revisited: Is simple beautiful? in 2012 Fourth international workshop on quality of multimedia experience. 2012. IEEE.

[24] Han Y., , A new image fusion performance metric based on visual information fidelity. Information fusion, 2013. 14(2): p. 127–135.

[25] Brunet D., Vrscay E.R., and Wang Z., On the mathematical properties of the structural similarity index. IEEE Transactions on Image Processing, 2011. 21(4): p. 1488–1499. 22042163 10.1109/TIP.2011.2173206

[26] Verma R., , MoNuSAC2020: A multi-organ nuclei segmentation and classification challenge. IEEE Transactions on Medical Imaging, 2021. 40(12): p. 3413–3423. 34086562 10.1109/TMI.2021.3085712

[27] Lotz J., , Comparison of consecutive and restained sections for image registration in histopathology. Journal of Medical Imaging, 2023. 10(6): p. 067501–067501. 38074626 10.1117/1.JMI.10.6.067501 PMC10704256

[28] Paknezhad M., , Regional registration of whole slide image stacks containing major histological artifacts. BMC bioinformatics, 2020. 21(1): p. 558. 33276732 10.1186/s12859-020-03907-6 PMC7718714

[29] Low D.G., Distinctive image features from scale-invariant keypoints. Journal of Computer Vision, 2004. 60(2): p. 91–110.

[30] Clifton K., , STalign: Alignment of spatial transcriptomics data using diffeomorphic metric mapping. Nature communications, 2023. 14(1): p. 8123. 10.1038/s41467-023-43915-7 PMC10709594 38065970

[31] Radford A., Learning transferable visual models from natural language supervision. in International conference on machine learning. 2021. PMLR.

